# Does periphyton turn less palatable under grazing pressure?

**DOI:** 10.1093/ismeco/ycae146

**Published:** 2024-11-19

**Authors:** Feng Zhu, Xiang Tan, Xingzhong Wang, Quanfa Zhang

**Affiliations:** Key Laboratory of Aquatic Botany and Watershed Ecology, Wuhan Botanical Garden, Chinese Academy of Sciences, Wuhan 430074, P.R. China; Key Laboratory of Chemistry in Ethnic Medicinal Resources, Yunnan Minzu University, Kunming 650031, P.R. China; Key Laboratory of Aquatic Botany and Watershed Ecology, Wuhan Botanical Garden, Chinese Academy of Sciences, Wuhan 430074, P.R. China; Danjiangkou Wetland Ecosystem Field Scientific Observation and Research Station, The Chinese Academy of Sciences & Hubei Province, Wuhan 430074, P.R. China; Zhejiang Provincial Key Laboratory of Aquatic Resources Conservation and Development, College of Life Sciences, Huzhou University, Huzhou 313000, P.R. China; Key Laboratory of Aquatic Botany and Watershed Ecology, Wuhan Botanical Garden, Chinese Academy of Sciences, Wuhan 430074, P.R. China; Danjiangkou Wetland Ecosystem Field Scientific Observation and Research Station, The Chinese Academy of Sciences & Hubei Province, Wuhan 430074, P.R. China

**Keywords:** biofilm, food webs, food quality, fatty acids, top-down, transcriptome analysis

## Abstract

Periphyton acts as an important primary producer in stream food webs with bottom-up grazing pressure and is also subject to effects of top-down grazing pressure. However, the underlying mechanisms of these interactions remain unclear. In this study we conducted a mesocosm experiment to explore the periphyton response to grazing pressure by the freshwater snail *Bellamya aeruginosa* in relation to food quality indicated by polyunsaturated fatty acid (PUFA) biomarkers, including eicosapentaenoic acid (20:5n3) and the 22C fatty acid docosahexaenoic acid (22:6n3), which are essential for cell growth and reproduction and cannot be synthesized by most consumers of periphyton. Results indicated that periphyton grazing pressure led to a decrease in *Bacillariophyta*, which contain high-quality PUFAs such as eicsapentaenoic acid and docosahexaenoic acid, and an increase in *Cyanophyta* and *Chlorophyta*, which are rich in 18C PUFAs such as linoleic acid (18:2n6) and alpha-linolenic acid (18:3n3). We observed upregulation of genes that participate in lipid metabolism promoting unsaturated fatty acid biosynthesis, alpha-linolenic acid metabolism, and glycerophospholipid metabolism, which are related to the carbohydrate and energy metabolism maintaining the energy stability of periphyton. These results demonstrate that the food quality of periphyton decreased under grazing pressure and also elucidate the compositional, chemical, and molecular perspectives of the interactive bottom-up and top-down effects on structuring stream food webs.

## Introduction

The river ecosystem is one of the most important ecosystems on earth and provides valuable habitats for diverse microbes that interact as food webs [1–3]. The benthic food web is a crucial component of river ecosystems [4, 5], presenting the intricate flow of energy and nutrients from one species to another [6, 7]. Structurally, the benthic food web is limited by light and nutrients, i.e., bottom-up effects, and also controlled by consumers, i.e., top-down effects [8–10]. The complex networks between the benthic microbes and their consumers create a web of interconnections, and the web sustains the diverse and dynamic microbes in the aquatic ecosystems [2, 11].

Periphyton, a basal resource of the benthic food web, is composed of a variety of autotrophic (e.g., benthic algae) and heterotrophic (e.g., fungi) microbes and benthos [12–14]. Benthic algae utilize dissolved inorganic carbon, converts it into organic matter [15–17], and enables transfer of energy from primary producers to microbes of higher trophic levels to maintain ecosystem processes, including productivity [12, 14], which play critical roles in the energy flow of riverine ecosystems [18–20]. Moreover, periphyton is considered a high-quality food resource for consumers due to its high concentrations of polyunsaturated fatty acids (PUFAs) [14]. The biomass and structure of PUFAs are strongly affected by grazing from primary consumers, and in higher trophic levels these are preyed upon by secondary consumers such as badgers and river otters [20–22].

Although PUFAs are essential for maintaining cell growth and reproduction, most consumers cannot synthesize them or have limited ability to do so [23, 24]. Additionally, significant differences exist among the contents and types of fatty acids found in different microorganisms in periphyton. Diatoms (*Bacillariophyta*) are eukaryotic autotrophs and are normally 2 to 200 μm in size. *Bacillariophyta* are usually rich in omega-3 fatty acids, such as eicosapentaenoic acid (EPA) and docosahexaenoic acid (DHA), which are high-quality foods for consumers, whereas bacteria mainly contain 16C and 18C saturated fatty acids (SAFAs) and 18C monounsaturated fatty acids (MUFAs) [25–27]. Consumers often prioritize the intake of PUFA-rich foods to optimize their nutrient intake and maintain a balanced element composition [24, 28]. This selective feeding may reduce the number of species with high PUFA abundance in the periphyton community or lead to their replacement by other species better adapted to the grazing environment [29] and the initiation of a chain reaction in the aquatic food webs [30–32].

This strength of prey–predator interactions and their importance for ecosystem functioning is well demonstrated in multiple ecosystems [33–36], e.g., predator and prey biomass in freshwater, marine, and terrestrial ecosystems follow a general scaling law with exponents consistently near ¾ [35, 36], and such interactions significantly influence material and energy transfer to higher trophic levels [37, 38]. Nevertheless, the precise molecular pathways, including transcription of enzymes, underlying these changes and their modulation remain largely elusive. Transcriptome-derived RNA sequencing (RNA-seq) is the sum of all RNA transcribed by a particular tissue or cell at a certain stage of development or functional state [39, 40] and consequently reveals the molecular mechanisms of specific biological processes from a holistic perspective [41].

In this study we conducted a mesocosm experiment to investigate the prey–predator interactions between benthic invertebrates and periphyton, in particular responses of periphyton to grazing pressure related to food quality (determined on the basis of PUFAs) in compositional, biochemical, and RNA-seq transcriptomics perspectives. We identified the differences in community composition in periphyton and major biochemical metabolic fatty acid and signal transduction pathways related to differential gene expressions and highlighted the association between grazing pressure exerted by consumers and variation in the palatability (also associated with PUFA profiles) of periphyton. Ultimately, we aimed to reveal the multidimensional mechanisms at community and molecular levels that govern the transformations in the food quality of periphyton when subjected to grazing pressure.

## Materials and methods

### Mesocosm experiment setup

We performed a 4-week manipulative experiment with a facility/mesocosm in a greenhouse in the Wuhan Botanical Garden (Wuhan, China; 30°30′N, 114°31′E) from November to December 2020. The facility was composed of 10 fully flow-through mesocosms (volume, 1000 L; diameter, 1.2 m; height, 0.8 m). Each mesocosm was filled with 500 l water, and an aquarium pump was installed in each mesocosm to circulate water (Fig. S1). Water was transported back from the Chuka River, Macheng, Hubei Province, China (31°5′N115°18′E), which is ~140 km away from the Wuhan Botanical Garden, Chinese Academy of Sciences.

In each equipment setup the bottom was covered with periphyton cobbles (also called biofilms) of 15–20 cm in diameter. Rocks (cobbles) from the Chuka River were also collected and transported to the laboratory. After the visible macrozoobenthos had been carefully removed with forceps, the cobbles were taken to a laboratory and then cultured for 1 week to adapt to experimental conditions.

The 10 sets of mesocosms were divided into two groups, one group (Grazed) with a consumer population of freshwater snails (*Bellamya aeruginosa*) at a density of 80/m^2^ [42], and another group (Ambient) as the control (Fig. S1). *Bellamya aeruginosa* is dioecious and ovoviviparous and ubiquitously distributed in streams, ponds, reservoirs, and other freshwater bodies [43]. It is also an important species of the benthic macroinvertebrate and the dominant benthic species in many waterways [21, 28]. *Bellamya aeruginosa* snails are easy to breed and have moderate size, fast growth, and strong reproductive ability and were commercially obtained in a local aquarium shop.

The water temperature at the beginning of the study was 12°C–15°C, the pH was 8.57, nitrate–nitrogen was 0.76 mg/l, and dissolved oxygen was 10.95 mg/l. Periphyton, *Bellamya aeruginosa,* and water samples were taken on days 0, 7, 14, and 21 for further analysis.

### Epilithic algal community

We randomly selected six to eight cobbles from each of the mesocosms and scraped the stones with a hard-bristled toothbrush. Epilithic algae were rinsed several times with distilled water to ensure that periphyton was brushed off completely. The periphyton samples from six to eight cobbles were pooled to one sample and then were kept to one 50-ml centrifuge tube per mesocosm. In addition, samples were diluted using MilliQ water (15 ml) and fixed with Lugol’s solution (1:50 dilution) [44]. Lugol’s solution is a solution of elemental iodine (5%) and potassium iodide (10%) [45]. *Bacillariophyta*, *Cyanophyta*, *Chlorophyta*, and other epilithic algae were identified and counted by use of the methods described by Hu and Wei [46] and Hu et al. [47]. The composition of the community was determined using an inverted microscope (Olympus BX51, Olympus Corporation, Tokyo, Japan). Before identification and counting, samples were thoroughly mixed, and then 0.1 ml of sample (after pretreatment) was put into a 0.1-ml counting frame by use of a pippette and counted under a 10 × 40 microscope. In total, 100 fields of view at a minimum and 3 replicates of each sample were counted.

### Lipid extraction and fatty acid analysis

Lipids were extracted according to a modified version of the method proposed by Vesterinen et al. [14]. Samples (periphyton and *Bellamya aeruginosa*) for FA analysis were freeze dried (FreeZone 4.5 l) and homogenized and then reweighed after freeze drying. Lipids were extracted from the lyophilized and homogenized samples using a 15-ml mixture of 0.2 M KOH:MeOH (1:1) maintained at 50°C for 60 minutes [48], and then the nonlipid material was removed by sonication, vortex, and centrifugation. Those steps were repeated three times. Next, to form FA methyl esters (FAMEs), a methanolic sulfuric acid (1:100) mixture and toluene were added to the lipid extract and the solution was incubated in a water bath at 50°C for 16 hours. FAMEs were analyzed by gas chromatography–mass spectrometry (Agilent 5975C) equipped with HP-88 columns (100 m × 0.25 mm, 0.20 μm, Agilent, USA). Helium (constant flow: 1.2 ml/min) was used as the carrier gas with an injection volume of 1 μl under a split ratio of 25:1. Identification of individual FAMEs was based upon comparing the mass-to-charge ratio and peak area with that of standard FAME mixtures (mixtures of 37 FAs, a FAME mixture; 47 885-U Supelco) and the National Institute of Standards and Technology database to determine relative FAME content. The FA component was expressed as a percentage relative to the total FA.

### Sampling for RNA analysis

Total RNA was extracted from periphyton samples by TRIzol reagent kit (Beijing, Tiangen Biochemical Technology, China) with three replicates for each sample, and its concentration and purity were detected by Nanodrop 2000 (Thermo Scientific, Wilmington, USA). Agarose gel electrophoresis (1.5%) was used to detect the integrity of the RNA (whether there was dispersion or genomic DNA contamination). The RNA extracted from each sample was subsequently sent to Majorbio Bio-Pharm Technology Co., Ltd. (Shanghai, China) for sequencing. An Agilent2100 Bioanalyzer (Agilent Technologies, Santa Clara, USA) was used to determine RNA integrity number values. The library was constructed after the sample was tested as qualified. The poly(A) messenger RNAs (mRNAs) were enriched with oligo (dT) beads, and then fragmented to small pieces. These small mRNAs were converted to complementary DNA by reverse transcription, and after end repair, adapter ligation, and agarose gel electrophoresis filtration, polymerase chain reaction was carried out on the transcripts and sequenced using the Illumina HiSeq™ 2500 sequencing platform.

For quality control, the raw reads were quality controlled using SOAPnuke software (v 2.1.0) to obtain high-quality clean reads through image recognition, decontamination, joint removal, adapter sequence removal, ambiguous reads (“N”), and low-quality reads (reads with >10% N bases or bases with a quality score <20) [49–51]. *De novo* assembly was separately performed on the high-quality clean read datasets to obtain unigenes using Trinity software [52]. The unigenes were BLASTX (https://blast.ncbi.nlm.nih.gov/Blast.cgi) against the NCBI nonredundant protein database (NR), Swiss-Prot, Kyoto Encyclopedia of Genes and Genomes (KEGG), Clusters of Orthologous Groups (COGs), and Gene Ontology (GO) to derive protein function with an E-value threshold of 1.0E−5. The KEGG pathway annotation and GO and COGs functional classifications were also analyzed with each sample’s unigenes [53,54].

### Data analysis

All data were transformed for normal distribution approximation before analyses. Paired two-tailed *t*-tests were conducted to analyze the FA (%) data. The data were expressed as mean ± SE, and *P* < .05 indicated statistical significance. Eight individual FA or groups of FA were derived to represent essential FAs.

In transcriptome data, the DEGs were identified using the DEGSeq2 (v1.6.3) based on reads per kilobase transcriptome per million mapped reads [55,56], with *P*-adjusted < .05 and a log 2FC| > 2 setting as the threshold to indicate significant differential expression [57,58]. Significant DEG output was further enriched by GO and KEGG annotation. Enrichment was measured by Rich factor, Q value, and gene number. The ratio of the number of DEGs annotated in a pathway term to the number of all genes annotated in the same pathway term was defined as the Rich factor. The Q value is the *P* value corrected by the multiple hypothesis tests, and its value range was between 0 and 1. The GO term and KEGG Pathway with Q value ≤ .05 was selected to plot the GO/KEGG enrichment bubble diagram of DEGs. All data analysis was performed with statistical software R version 4.0.2 (R core team, 2020).

## Results

### Environmental variables and algal composition in periphyton

**Figure 1:**
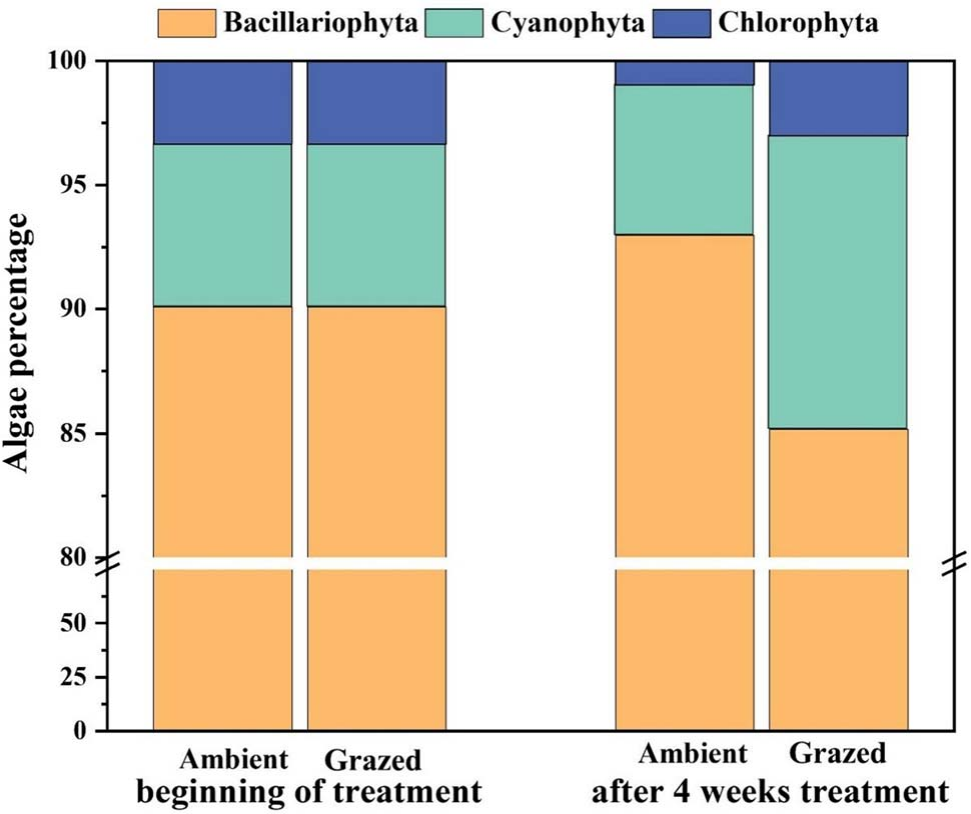
Proportion of *Bacillariophyta*, *Chlorophyta*, and *Cyanophyta* in algal community in periphyton at the beginning and after 4-week treatment. Ambient, without consumer *Bellamya aeruginosa*; Grazed, with consumer *Bellamya aeruginosa* addition.

At the start and after 4 weeks, environmental variables remained stable. Following the Grazed treatment, physical and chemical parameters showed no significant changes, with the temperature at 10°C, pH at 8, and dissolved oxygen at 11 mg/l (Table S1). In both the Ambient and Grazed groups, diatoms were dominant and accounted for more than 85% of organisms. Initially, the benthic algal community was dominated by *Bacillariophyta* (90.07%) with smaller percentages of *Cyanophyta* (6.58%) and *Chlorophyta* (3.35%). After 4 weeks, the proportion of *Bacillariophyta* in the benthic algal community in periphyton decreased and the proportion of *Cyanophyta* increased in the Grazed treatment compared to the Ambient group (Fig. 1). There was an increase in the proportion of *Bacillariophyta*, which accounted for 92.97% in the Ambient group after the 4 weeks. In comparison, *Cyanophyta* decreased, accounting for 6.06%. In contrast, the Grazed treatment group exhibited a decrease in *Bacillariophyta* (85.17%) and an increase in *Cyanophyta* (11.80%), while *Chlorophyta* remained relatively stable (~3%). Also, after a 4-week treatment there were significant differences between the Grazed and Ambient groups of about 22%, 160%, and 317% in *Bacillariaphyta*, *Cyanophyta*, and *Chlorophyta*, respectively (Table S2). The taxa identified in periphyton are shown in Table S3.

### FA profiles in periphyton

**Figure 2:**
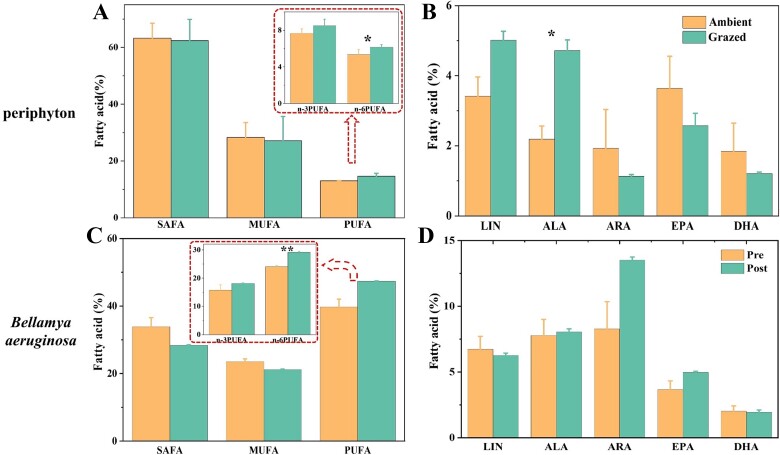
Main groups of FAs and five specific FAs (percentages of the total FAs, mean ± SE) in periphyton (A, B) in Ambient (without *Bellamya aeruginosa*) or Grazed (with *Bellamya aeruginosa*) groups, and in *Bellamya aeruginosa* (C, D) after 4 weeks of treatment. SAFAs, MUFAs, and PUFAs can be categorized to n-3PUFA and n-6PUFA. LIN, 18:2n6c; ALA, 18:3n3; ARA, 20:4n; EPA, 20:5n3; DHA, 22:6n3.. ALA, EPA, and DHA are n-3 PUFAs; LIN and ARA are n-6 PUFAs. LC-PUFAs contain ARA, EPA, and DHA. ALA and LIN are 18C PUFAs. ^*^P < .05, ^**^P< .01, ^***^P < .001.

A total of 20 fatty acids were extracted and determined from periphyton (Table 1). The addition of grazers resulted in a decrease in SAFAs and MUFAs, and an increase in PUFAs in periphyton after 4 weeks of treatment. The n-6 PUFAs linoleic acid (LIN, 18:2n6c) and arachidonic acid (ARA, 20:4n6) increased by 0.8% with consumer addition (Fig. 2A). The percentage of 18C fatty acids (LIN, 18:2n6c and alpha-linolenic acid [ALA] 18:3n3) also increased, while 20C fatty acids (ARA 20:4n6 and EPA 20:5n3) and 22C fatty acids (DHA 22:6n3) decreased in periphyton (Fig. 2B).

**Table 1:** Fatty acid composition in periphyton at the after 4 weeks of treatment.

	Percentages relative to total fatty acids, mean ± SE	
Fatty acid	Ambient	Grazed
SAFA		
C12:0	0.48 ± 0.19	0.48 ± 0.29
C13:0	2.03 ± 1.60	1.16 ± 0.20
C14:0	3.37 ± 2.15	6.43 ± 0.90
C15:0	0.61 ± 0.11	0.84 ± 0.15
C16:0	40.45 ± 6.31	39.72 ± 5.73
C17:0	1.41 ± 0.06	0.74 ± 0.11
C18:0	3.84 ± 0.35	4.66 ± 0.52
C20:0	1.55 ± 0.70	0.70 ± 0.07
C22:0	1.40 ± 0.07	2.41 ± 0.07
C24:0	3.52 ± 1.04	1.08 ± 0.20
MUFA		
C14:1	1.35 ± 0.98	2.19 ± 2.00
C16:1	18.28 ± 3.80	9.93 ± 8.56
C17:1	1.23 ± 1.04	0.23 ± 0.01
C18:1n9t	1.91 ± 1.64	3.49 ± 3.20
C18:1n9c	5.56 ± 5.33	11.3 ± 1.24
PUFA		
LIN C18:2n6c	3.41 ± 0.55	5.01 ± 0.26
ALA C18:3n3	2.19 ± 0.37	4.72 ± 0.31
ARA C20:4n6	1.93 ± 1.11	1.13 ± 0.05
EPA C20:5n3	3.64 ± 0.92	2.57 ± 0.35
DHA C22:6n3	1.84 ± 0.81	1.21 ± 0.04

The fatty acid profiles of *Bellamya aeruginosa* also changed. Specifically, the proportions of SAFAs and MUFAs decreased by 5.49% and 2.37%, respectively, while PUFAs increased by 7.58% (Fig. 2C). Notably, n-6 PUFA increased by 5.16% (Fig. 2C), with ARA (20:4n6) and EPA (20:5n3) increasing by 5.22% and 1.31%, respectively (Fig. 2D).

### RNA-Seq datasets

Approximately 66.4 and 59.9 million clean read pairs were obtained from the Ambient and Grazed treatment groups, respectively (Table S4). A total of 803 356 transcripts were annotated (Table S5), with more than 30% of them being annotated with GO (32.63%) and KEGG (29.56%) and more than 25% of them annotated into KOG (27.92%).

Unigenes were observed in all biological physiological processes (Fig. S2). Of these, translation, ribosome structure, and biogenesis were annotated in a large number. In addition, about 1 000 (4.46%) unigenes in lipid transport and metabolism were annotated. Genes allocated in GO showed that the cellular component (CC) had the largest number of genes (731 538), followed by biological process (653 922), and molecular function (359 214) (Fig. S3). A total of 112 025 transcripts were related to metabolism, of which energy metabolism and carbohydrate metabolism were 22 780 and 20 866, respectively. Amino acid metabolism and lipid metabolism followed closely with 14 862 and 9 457, respectively (Fig. S4).

### DEG metabolic pathways (KO and KEGG pathways)

**Figure 3:**
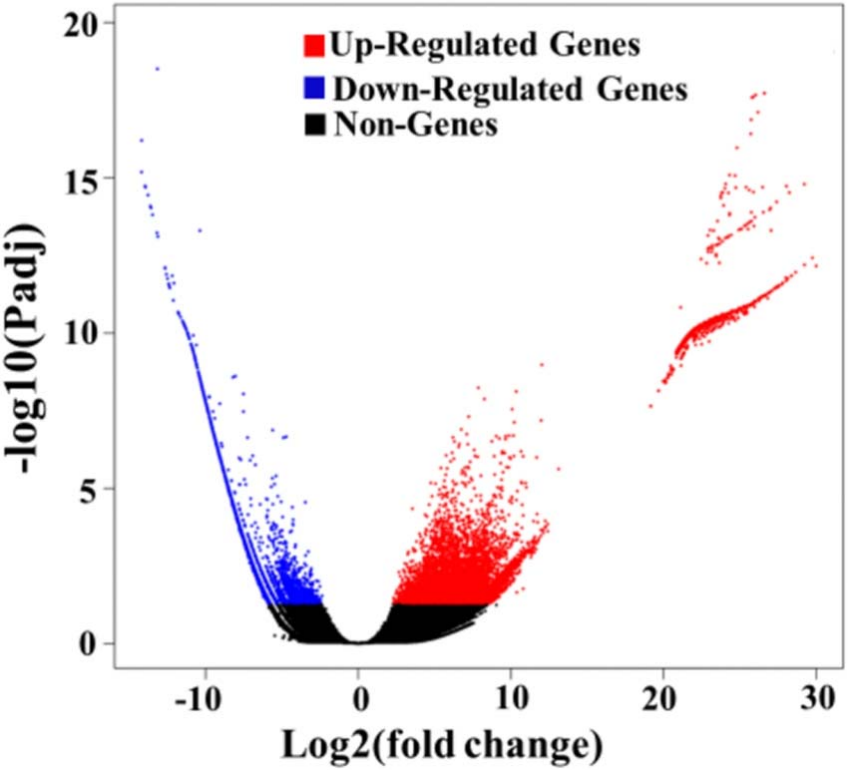
Volcano map of DEGs. The horizontal axis indicates expression changes (log) of the genes in different treatments while the vertical axis shows the differences of gene expression. Splashes are for different genes. Dots represent genes with no discrepancy (black), significant upregulation (red) and downregulation (blue).

There were 14 478 unigenes with significantly differential expression between the Ambient and Grazed treatment with GO annotation (Fig. 3). Among them, 10 301 (71.1%) unigenes were upregulated and 4 177 (28.9%) were downregulated. The principal biological functions with DEGs were related to cellular components and biological processes (Fig. S5).

In total 7 339 DEGs were annotated into 128 KEGG pathways, of which 5 626 (76.7%) were upregulated and 1 713 (23.4%) were downregulated under grazing pressure (Fig. 3). These DEGs could be classified into 10 categories, including lipid metabolism, carbohydrate metabolism, energy metabolism, and membrane transport. Under lipid metabolism, pathways that were significantly affected (by Q value) were alpha-linolenic acid (ALA) metabolism (ko00592), biosynthesis of UFA (ko01040), glycerophospholipid metabolism (ko00564), arachidonic acid metabolism (ko00590), and fatty acid degradation (ko00071) with significant upregulation (Fig. S6).

**Figure 4:**
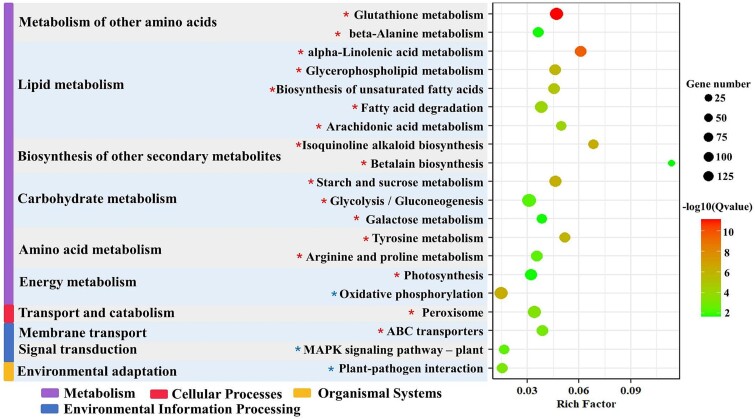
Bubble diagram of KEGG pathway enrichment analysis of DEGs. The vertical axis indicates the KEGG pathway and the horizontal axis represents the Rich factor. The size of the bubbles indicates the number of genes in the KEGG pathway. Pathways that are significantly upregulated (red bubbles) and significantly downregulated (green bubbles).

Under carbohydrate metabolism, starch and sucrose metabolism (ko00500), glycolysis/gluconeogenesis (ko00010) and galactose metabolism (ko00052) were upregulated. Genes involved in photosynthesis (ko00190) within the energy metabolism pathway were also upregulated. It is noteworthy that the two pathways, i.e., transport and catabolism and membrane transport, showed a higher number of upregulated DEGs, including those related to peroxisome (ko04146) and ATP-binding cassette transporters (ko02010). Also, the transport and catabolism and membrane transport pathways showed a higher number of upregulated DEGs and contained those related to peroxisome (ko04146) and ATP-binding cassette transporters (ko02010) (Fig. 4).

**Figure 5:**
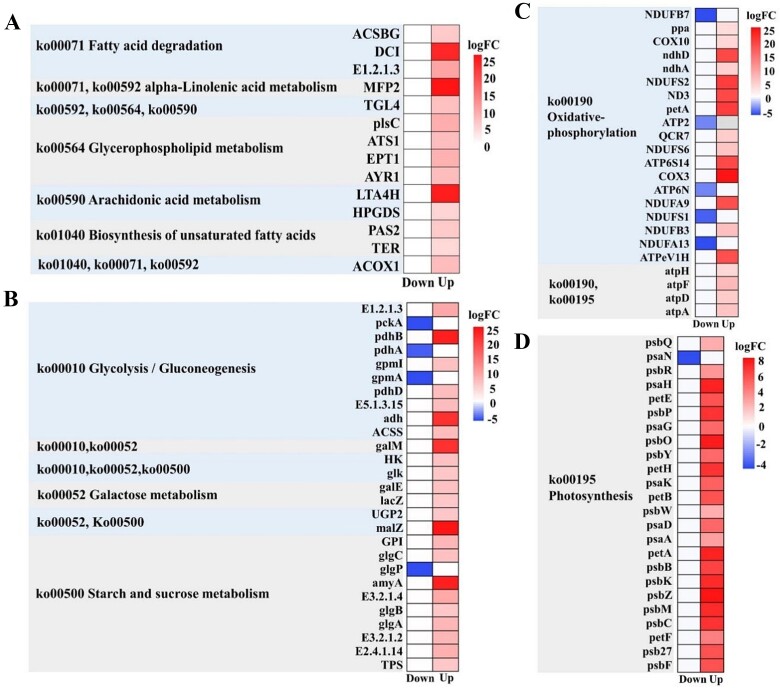
DEGs by predation pressure in periphyton. Lipid metabolic (A), carbohydrate metabolism (B), and energy metabolism (C, oxidative-phosphorylation and D, photosynthesis) pathways. High log2 of fold change numbers (red) indicate significant upregulation, while low log2 of fold change numbers (blue) indicate significant downregulation. (A), ACSBG, acyl-CoA synthetase; DCI, Enoyl-CoA hydratase; E1.2.1.3, retinal dehydrogenase; MFP2, enoyl-CoA hydratase; TGL4, triacylglycerol lipase; plsC, 1-acyl-sn-glycerol-3-phosphate acyltransferase; ATS1, glycerol-3-phosphate O-acyltransferase; EPT1, ethanolamine phosphotransferase; AYR1, predicted dehydrogenase; LTA4H, leukotriene-A4 hydrolase; HPGDS, hematopoietic prostaglandin D synthase; PAS2, very-long-chain (3R)-3-hydroxyacyl-CoA dehydratase; TER, very-long-chain enoyl-CoA reductase; ACOX1, acyl-coenzyme a oxidase. (B) E1.2.1.3, aldehyde dehydrogenase; glgB, 1,4-alpha-glucan-branching enzyme; ACSS, acyl-CoA synthetase; galM, aldose 1-epimerase; amyA, alpha-amylase; malZ, alpha-glucosidase; TPS, alpha-trehalose-phosphate synthase; lacZ, beta-galactosidase; E3.2.1.4, endoglucanase; glk, glucokinase; glgC, glucose-1-phosphate adenylyltransferase small subunit; GPI, glucose-6-phosphate isomerase; glgP, glycogen phosphorylas; glgA, starch synthase; HK, hexokinase-6; E3.2.1.2, lysosomal alpha-mannosidase; pckA, phosphoenolpyruvate carboxykinase; gpmI/a, phosphoglycerate mutase; E2.4.1.14, sucrose-phosphate synthase; E5.1.3.15, putative glucose-6-phosphate 1-epimerase OS; pdhA/B/D, pyruvate dehydrogenase; adh, tRNA-specific adenosine deaminase; galE, UDP-glucose 4-epimerase 2; UGP2, UDP-glucose pyrophosphorylase. (c, d), ndhA, NDUF/B7S2/A9/A13/SI/B3, NADH:Ubiquinone oxidoreductase; psbY, photosystem II core complex proteins; psbM, photosystem II reaction center protein; ATPeV1H, V-type proton ATPase; petA/B, cytochrome; psbF, cytochrome b559 subunit beta; COX3, cytochrome c oxidase subunit; atpA/D, F0F1-type ATP synthase; petF, ferredoxin; COX10, Heme a farnesyltransferase; atpF/H, mitochondrial F1F0-ATP synthase; ndhD, NAD (P) H-quinone oxidoreductase chain; NDUFS6, NADH dehydrogenase; ND3, NADH–ubiquinone oxidoreductase chain; petH, NADP/FAD dependent oxidoreductase; psb Q/P/O, oxygen-evolving enhancer protein; psaA/N/D/K/G, photosystem I reaction center subunit; psbR/C/B/Z/K/W/27, photosystem II reaction center protein; ATP2, plasma membrane calcium-transporting ATPase; petE, plastocyanin; ppa, pyrophosphate-energized vacuolar membrane protonpump; QCR7, ubiquinol cytochrome c reductase; ATP6N/S14, V-type proton ATPase.

**Figure 6:**
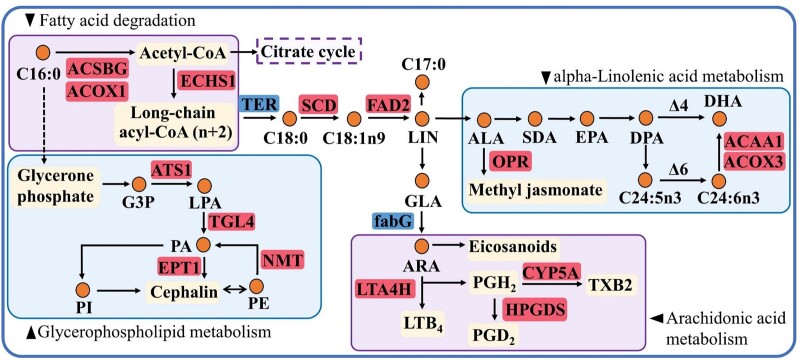
Simplified correlations between alpha-linolenic acid metabolism, biosynthesis of unsaturated fatty acids, glycerophospholipid metabolism, arachidonic acid metabolism ,and fatty acid degradation under predation pressure conditions. Expression of statistically significant DEGs associated with the respective pathways are highlighted based on KEGG pathway modules. Significantly upregulated genes (red) and downregulated genes (blue) are displayed. ACAA1, 3-ketoacyl-CoA thiolase; ACOX1 and ACOX3, acyl-coenzyme a oxidase; ATS1, glycerol-3-phosphate O-acyltransferase; CYP5A, cytochrome; ECHS1, enoyl-CoA hydratase; ELO3, fatty acid elongase 3; EPT1, ethanolamine phosphotransferase; FAD2, Delta (12) fatty acid desaturase; fabG, estradiol 17-beta-dehydrogenase 8; HPGDS, hematopoietic prostaglandin D synthase; KAR, very-long-chain 3-oxoacyl-CoA reductase; KCS, 3-ketoacyl-CoA synthase; LTA4H, leukotriene-A4 hydrolase; NMT, phosphoethanolamine N-methyltransferase; OPR,12-oxophytodienoic acid reductase; PCYT1, choline-phosphate cytidylyltransferase; PHS1, very-long-chain (3R)-3-hydroxyacyl-CoA dehydratase.; SCD, acyl-CoA desaturase (fragment); TER, very-long-chain enoyl-CoA reductase; TGL4, triacylglycerol lipase; G3P, glycerol-3-phosphate; LPA, lysophosphatidate; PA, phosphatidic acid; PI, phosphatidylinositol; PE, phosphatidyl ethanolamine.

**Figure 7:**
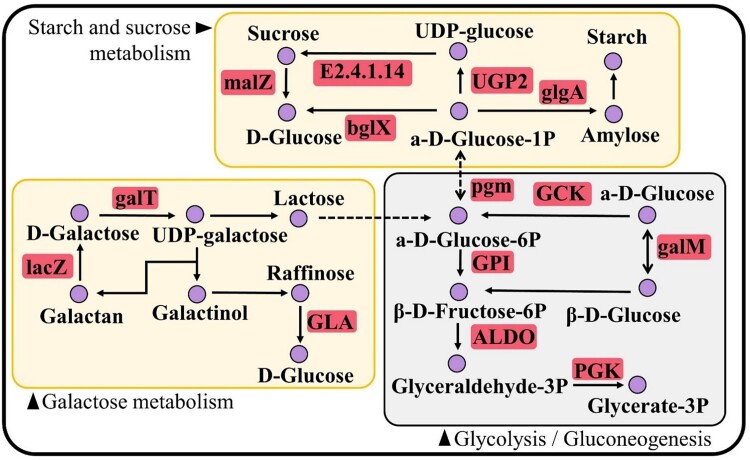
Simplified correlations between starch and sucrose metabolism and glycolysis and galactose metabolism under predation pressure. Expression of all statistically significant DEGs associated with the respective pathways are highlighted. Significantly upregulated genes (red) and downregulated genes (blue) are displayed. ALDO, fructose-bisphosphate aldolase; bglX, beta-glucosidase; E2.4.1.14, sucrose-phosphate synthase; galM, aldose 1-epimerase; GEB1, 1,4-alpha-glucan branching enzyme; glgA, starch synthase; GPI, glucose-6-phosphate isomerase; lacZ, beta-galactosidase; malZ, alpha-glucosidase; PGK, phosphoglycerate kinase; pgm, phosphoglucomutase; galT, hexose-1-phosphate uridylyltransferase; GLA, alpha-galactosidase; GCK, glucokinase; UGP2, UDP-glucose pyrophosphorylase.

**Figure 8:**
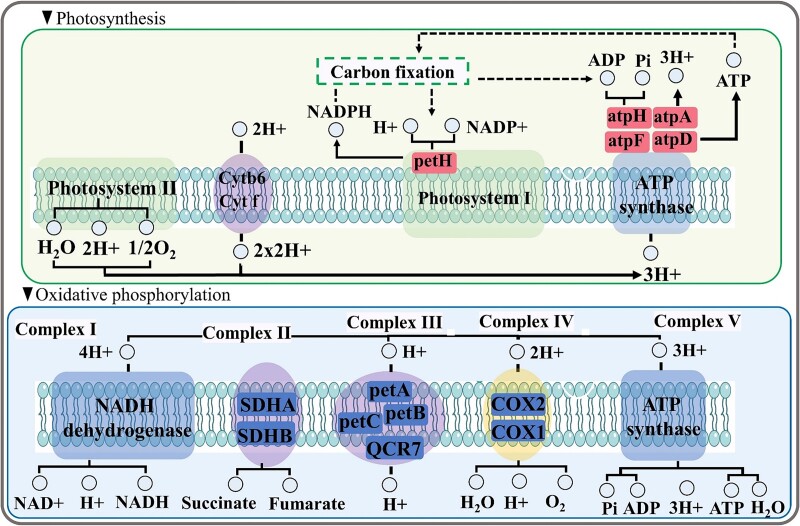
Expression of DEGs associated with the photosynthesis and oxidative phosphorylation under predation pressure. The location of putative genes encoding light harvesting complexes, photosystems I and II, and ATP synthase are highlighted based on KEGG pathway modules. Significantly upregulated genes (red) and downregulated genes (blue) are displayed. petH, NADP/FAD dependent oxidoreductase; atpA/D/F/H, F0F1-type ATP synthase; COX1/2, cytochrome c oxidase subunit; PetA/B/C, cytochrome; SDHA/B, succinate dehydrogenase (ubiquinone) flavoprotein subunit.

Genes from lipid metabolism, carbohydrate metabolism, and energy metabolism pathways exhibited different expression levels between the grazed transcriptome and the ambient transcriptome datasets (Fig. 5–8). Genes related to FA metabolism, including biosynthesis of unsaturated FA, were mostly upregulated (Fig. 5A, Fig. 6). Some important genes involved in the FA degradation pathway, such as acyl-CoA synthetase (ACSBG) and enoyl-CoA hydrolase (DCI), as well as retinal dehydrogenase (E1.2.1.3), were upregulated (Fig. 5A, Fig. 6). At the same time, some important genes involved in the metabolism of alpha-linolenic acid (ALA) and ARA, such as enoyl-CoA hydrolase (MFP2) and leukotriene-A4 hydrolase (LTA4H), as well as triglyceride lipase (TGL4) and hematopoietic prostaglandin D synthase (HPGDS), were also upregulated. Under grazing pressure, estradiol 17-beta-dehydrogenase 8 (fabG) was significantly downregulated, leading to reduced extension of GLA (gamma-linolenic acid, 18:3n6) to ARA (20:4n6). Genes related to the biosynthesis of unsaturated FAs, such as very long-chain (3R)-3-hydroxyacyl-CoA dehydratase (PAS2) and very long-chain enoyl-CoA reductase (TER) (Fig. 5A), were upregulated. Another factor promoting FA accumulation can be observed through the upregulation of several glycerol phospholipid genes related to glycerol-3-phosphate O-acyltransferase (ATS1), ethanolamine phosphotransferase (EPT1), and predicted dehydrogenase (AYR1) (Fig. 5A, Fig. 6).

The expression of glycerol-3-phosphate O-acyltransferase (ATS1) increased significantly under graze pressure (Fig. 6). This enzyme catalyzes the esterification of long-chain fatty-acyl coenzyme-A to glycerol-3-phosphate (G3P), which results in the production of lysophosphatidate (LPA). LPA was then further metabolized to phosphatidic acid (PA) by the action of triacylglycerol lipase (TGL4). The conversion of PA to PI and PE was accelerated and ultimately leads to an increase in carbohydrate metabolism, indicating by the upregulation of starch and sucrose metabolism, glycolysis/gluconeogenesis, and galactose metabolism (Fig. 7).

Pyruvate served as a crucial precursor for the accumulation of intracellular FAs. The upregulation of pyruvate dehydrogenase (pdhB) facilitated the conversion of pyruvate to acetyl CoA (Fig. 5B). Interestingly, under grazing stress conditions, phosphoenolpyruvate carboxykinase (pckA) and phosphoglycerate mutase (gpmA) were downregulated in the glycolytic pathway. Concurrently, grazing stress induced a downregulation trend of glycogen phosphorylase (glgP) in the starch and sucrose metabolism pathway. The upregulation of α-amylase (amyA) (Fig. 5B) represented a crucial step in starch and sucrose metabolism, leading to a sustained increase in the activity of α-glucosidase (malZ) and aldose-epimerase (galM) (Fig. 5B, Fig. 7).

Genes responsible for the “photosynthesis” (ko00195) and “oxidative phosphorylation” (ko00190) pathways exhibited differential responses between the two treatment transcriptome datasets (Fig. 5C and D, Fig. 8). The putative transcripts encoding the active sites of ATP formation (atpA, atpD, atpF, and atpH) played a role in ATP synthase, while most NADH:ubiquinone oxidoreductases (NDUF/B7S2/A9/A13/SI/B3) were downregulated. Simultaneously, genes directly involved in Photosystem I (PSI), namely photosynthetic electron transport (petH), were upregulated. Additionally, while some genes related to PSI, such as psaN, were slightly downregulated, the core intrinsic proteins of Photosystem II (psbR, psbC, psbB, psbZ, psbK, psbW, psb27) were significantly upregulated (Fig. 5Cand D). However, metabolic potential involved in oxidative phosphorylation, such as succinate dehydrogenase A (SDHA) and SDHB, fumarate reductase, and F-type ATPase were downregulated under grazing pressure (Fig. 8).

## Discussion

Periphyton in streams exhibits high levels of heterogeneity in terms of spatial and temporal variations in basal resources [3,4,59], and predators might further affect their structure and function [60]. Under natural growth without consumer *Bellamya aeruginosa*, the total density of benthic algae decreased 4 weeks later (Fig. 1). It might be that the nutrients that algal growth depends on in waters were limited, resulting in reduced productivity and consequently in total biomass [61]. In contrast, the total density of benthic algae increased after the *Bellamya aeruginosa* addition, implying that exposure of algae to grazing pressure induces physiological stress responses [62]. Consumer foraging of benthic algae may have enhanced the energy transfer efficiencies from algae to higher trophic consumers [23,63], increased periphyton heterogeneity, and allowed algae to grow in large quantities in a short period of time.

Primary consumers possess an ability to assimilate long-chain polyunsaturated FAs (LC-PUFAs), as a food resource, particularly those rich in FAs, e.g., ARA, EPA, and DHA [64], affecting the biomass of microbe with high food quality in periphyton [22]. We found that the proportion of *Bacillariophyta* decreased and *Cyanophyta* increased in periphyton after four weeks of consumer added (Fig. 1), suggesting the preference of the consumer (*Bellamya aeruginosa*) for high quality food *Bacillariophyta* with the increase of PUFA by 7.58% (Fig. 2C). *Bacillariophyta* are a rich source of essential LC-PUFA, important for growth and development, reproductive behavior, and hormonal regulation for benthic animals [65], while *Cyanophyta* and *Chlorophyta* generally lack LC-PUFA [66–68]. As a result, consumers such as *Bellamya aeruginosa* are attracted to high-quality *Bacillariophyta*, leading to a reduction in the proportion of *Bacillariophyta* in the periphyton. A feeding experiment also revealed that *Bellamya aeruginosa* did not feed on cyanobacteria even when a diet containing only cyanobacteria was supplied, which implied that the snails did not feed on cyanobacteria [69].

Diverse conditions not only modify the community structure of benthic algae in periphyton, but also to some extent, alter its biochemical composition [70,71], in which PUFAs of the algal community account for the palatability. The addition of *Bellamya aeruginosa* had a noticeable effect on the FA composition of the periphyton. These changes might be attributable to the increased grazing pressure on the periphyton, which resulted in a fourfold increase in *Chlorophyta* density. Specifically, the relative content of LIN (18:2n6c) and ALA (18:3n3) decreased, while the proportion of n-6 PUFAs increased (Fig. 2). *Chlorophyta* is rich in shorter chains of LIN (18:2n6c) [66], which is an essential precursor for other n-6 PUFA [27]. As a result, the increase in density of *Chlorophyta* led to an increase in the relative abundance of FA. ALA (18:3n3), as a structural substance and metabolic regulator, plays a crucial role in growth, cellular metabolism, and energy supply for movement [27]. Under grazing pressure, epilithic organisms increase the synthesis of ALA (18:3n3) to support their growth and metabolism, resulting in a higher relative abundance of this FA. Conversely, the relative abundance of high-quality FAs, EPA (20:5n3) and DHA (22:6n3), decreases, likely because their primary source- *Bacillariophyta* within the periphyton- declines, reducing the overall palatability of the periphyton. The grazers *Bellamya aeruginosa* have also accumulated ARA besides EPA and, DHA, and this was probably related with an immune defense response [72]. This finding agrees with other studies that show ARA metabolism in *Bellamya aeruginosa* changed when it faced with stress of toxic cyanobacteria [73].

When microbes are exposed to changing environmental conditions, and stress, they often adapt by regulating their lipid metabolism pathways, including multiple metabolic and hormonal signaling pathways [74]. In our study, lipid metabolism pathway in periphyton was significantly upregulated under grazing pressure (Fig. 4), such as ARA metabolism (ko00590) and biosynthesis of unsaturated FAs (ko01040). Additionally, in arachidonic acid metabolism pathway, 12-oxophytodienoic acid reductase (OPR) significantly upregulated, promoting the formation of methyl jasmonate from ALA (18:3n3). OPR is a signaling molecule involved in defense and stress responses [75]. The addition of grazers further stimulated the effect of OPR and reduced the biomass extending from ALA (18:3n3) to SDA (18:4n3) in FAs. SDA extends and saturates to EPA and DHA, and consequently, the relative content of EPA and DHA was also reduced.

Lipid metabolic includes FA synthesis, transport of FAs into and out of plastids, and their binding to different classes of lipids [76]. Under grazing pressure, the upregulation of Acyl-CoA desaturase (SCD) and FA desaturase (FAD2) drives the conversion of saturated to unsaturated FAs (Fig. 5), and such shifting carbon flux towards lipid synthesis may be a response to stressful conditions [77]. Unsaturated FAs regulate the fluidity of cell membranes and promote the growth and reproduction of somatic cells, and play a major role in maintaining the stability of the organism itself [24,78]. Under grazing pressure, the pathway of the biosynthesis of unsaturated FAs was significantly upregulated to meet the organism’s demands for stability and growth.

Starch and lipids are the two main substances for energy storage, and they are interconvertible by sharing common precursors [79,80]. In response to grazing pressure, under carbohydrate metabolism, the starch and sucrose metabolism (ko00500), glycolysis/gluconeogenesis (ko00010) and galactose metabolism (ko00052) was upregulated (Fig. 4). Certain genes involved in starch and sucrose formation, such as glucose-1-phosphate uridylyltransferase (UGP2), starch synthase (glgA), alpha-glucosidase (malZ), and beta-glucosidase (bglX) were significantly upregulated (Fig. 7). The upregulation of genes bglX and malZ allow for the conversion of polysaccharides like sucrose and glucoside to glucose, which can then accelerate carbohydrate metabolism. Glucose can be converted into acetyl-CoA, which is a precursor for FA synthesis. The upregulation of beta-galactosidase (lacZ) and alpha-galactosidase (GLA) also enhances the formation of pyruvate, turning the carbon source towards lipid biosynthesis. Therefore, the upregulation of these genes can promote the conversion of carbohydrates to lipids and may be a key factor in lipid accumulation, hence affecting food quality of periphyton for consumers.

Photosynthesis drives the conversion of light energy into lipid biosynthesis through carbon dioxide fixation; the NADPH and ATP produced by this process also provide cellular energy, thereby inducing lipid accumulation [81,82]. Importantly, supply and regulation of ATP and NADPH/NADH heavily influences lipid accumulation in microalgae [81]. Our study found that genes involved in photosynthesis (ko00190) within the energy metabolism pathway was also upregulated (Fig. 4). The photosynthetic potential of algae in periphyton, including both photosystem I and photosystem II, was significantly enhanced under grazing pressure, leading to an overall increase in the potentials synthesis of NADPH and ATP (Fig. 8). Photosynthetic electron transport (petH) and energy-producing potentials (F-type ATPase of atpA, atpD, atpF) were significantly upregulated. The enhanced metabolic potentials in NADP^+^ reductase and F-type ATPase could potentially increase the carbon fixation ability and organic carbon synthesis. The increase of the number of ribosomes suggests that there is a greater production of proteins taking place (Fig. 3), then the upregulation of genes involved in lipid metabolism, carbohydrate metabolism and energy metabolism are consistent with this phenomenon, as these pathways are involved in the production and storage of energy.

Overall, under grazing pressure, the content of high-quality FAs i.e., long-chain PUFAs including ARA, EPA, and DHA, decreases in periphyton, and periphyton turn less palatable as food source. This is the first evidence of decrease of high food quality in periphyton through the alterations in profiles of FAs. Secondly, the observed significant alterations in the expression of metabolic pathway-related genes suggest that consumer significantly affect the metabolic regulation of FAs in periphyton. The alterations in lipid metabolism allow periphyton to rapidly adapt to environmental changes [76,83]. These results have important implications for understanding the dynamics of carbon in aquatic ecosystems, including the variation in carbon quality that basal resources can provide through bottom-up pathways and the role of consumers in shaping community structure.

## Conclusion

Our study reveals that the addition of primary consumers/predators can make periphyton as a food source less palatable through alteration of community of algae and the regulation through transcriptomes of microbe in periphyton. The presence of primary consumers greatly affects com/position of algae in periphyton through the way that the proportion of *Bacillariophyta* rich in high-quality food resources decreases and *Cyanophyta* and *Chlorophyta* which is considered as low food quality increase. On the other hand, molecularly, grazing pressure leads to a significant upregulation of the lipid metabolic pathway of periphyton, especially in the biosynthesis of unsaturated FAs, alpha-linolenic acid (ALA) metabolism, ARA metabolism, and FA degradation and glycerophospholipid metabolism. Moreover, periphyton carbohydrate and energy metabolisms are also significantly upregulated to maintain the energy supply of periphyton under grazing pressure. The study's findings shed light on how consumers affect the food quality of periphyton, as well as how these microbes maintain stability when face grazing/predation pressure, and reveals the mechanism of energy transmission and transfer between producers and consumers in stream food webs.

## Supplementary Material

MS20241031_SupplyMater_XT_ycae146

## Data Availability

Raw sequence data is deposited in the NCBI archive under accession number PRJNA1182968. All other data are available on figshare: https://doi.org/10.6084/m9.figshare.27324357.
