# Supplementary material for: Does periphyton turn less palatable under grazing pressure?
Source: ISME Commun. 2024 Nov 19;4(1):ycae146. doi: 10.1093/ismeco/ycae146 (PMC11697170; doi:10.1093/ismeco/ycae146)
Supplement: MS20241031_SupplyMater_XT_ycae146 [file ms20241031_supplymater_xt_ycae146.docx]

Table S1 Physical and chemical variables in the manipulative experiment in mesocosm.

| **Group** | **At the beginning** | **After 4 weeks treatment** | |
| --- | --- | --- | --- |
|  |  | **Ambient** | **Grazed** |
| Temperature (°C) | 12.15 | 9.7±0.15 | 10.2±0.42 |
| pH | 8.57 | 8.66±0.04 | 8.38±0.18 |
| NH_4_^+^-N (mg/L) | 0.757 | 1.03±0.42 | 7.36±3.44 |
| DO (mg/L) | 10.95 | 12.32±0.34 | 11.73±0.06 |

NH_4_^+^-N ammonium nitrogen, DO dissolved oxygen.

Table S2 The density of three dominant phyla of algae in periphyton.

| **Three dominant phyla of algae in periphyton** | **Density of algae**  **at the beginning**  **(cells·m^-2^)** | | **Density of algae**  **after 4 weeks treatment**  **(cells·m^-2^)** | |
| --- | --- | --- | --- | --- |
|  | **Ambient** | **Grazed** | **Ambient** | **Grazed** |
| Bacillariophyta | 4.84×10^4^ | 4.84×10^4^ | 3.83×10^4^ | 4.69×10^4^ |
| Cyanophyta | 0.35×10^4^ | 0.35×10^4^ | 0.25×10^4^ | 0.65×10^4^ |
| Chlorophyta | 0.18×10^4^ | 0.18×10^4^ | 0.04×10^4^ | 0.17×10^4^ |

Table S3 The percentage of algal species in periphyton at the beginning and after 4 weeks in manipulative experiment.

| **Taxa of algae in periphyton** | **At the beginning** | **After 4 weeks treatment** | | | |
| --- | --- | --- | --- | --- | --- |
|  |  | **Ambient** | **Grazed** | | |
| ***Bacillariophyta*** |  |  | |  |  |
| *Achnanthes* | 0.32% | 0.22% | | - |  |
| *Cocconeis* | - | 0.66% | | 2.64% |  |
| *Cymatopleura* | 0.16% | - | | 0.16% |  |
| *Cymbella* | 52.80% | 29.81% | | 19.56% |  |
| *Denticula* | 0.16% | 0.22% | | - |  |
| *Epithemia adnata* | - | - | | 0.16% |  |
| *Fragilaria* | 5.87% | 21.20% | | 24.41% |  |
| *Gomphonema* | 20.61% | 31.72% | | 26.19% |  |
| *Gyrosigma* | 0.32% | - | | - |  |
| *Melosira granulata* | 1.94% | 2.88% | | 2.60% |  |
| *Melosira granulata* var. angustissima | 1.29% | - | | - |  |
| *Melosira sp.* | 1.29% | - | | - |  |
| *Melosira varians* | 1.77% | - | | - |  |
| *Navicula* | 0.16% | 1.33% | | 2.27% |  |
| *Nitzschia* | 1.21% | 0.66% | | 1.38% |  |
| *Rhoicosphenia abbreviata* | - | 0.44% | | 0.81% |  |
| *Surirella* | - | - | | 1.62% |  |
| ***Cyanophyta*** |  |  | | |  |
| *Anabaena* | 1.61% | - | | 2.03% |  |
| *Chroococcus* | 0.97% | - | | - |  |
| *Oscillatoria* | 4.30% | 5.53% | | 9.74% |  |
| *Planktolyngbya* | 0.81% | 3.32% | | - |  |
| *Raphidiopsis* | 0.81% | - | | - |  |
| ***Chlorophyta*** |  |  | | |  |
| *Ankistrodesmus acicularis* | - | 0.44% | | 0.57% |  |
| *Closterium* | 0.16% | - | | - |  |
| *Kirchneriella* | 0.16% | - | | - |  |
| *Pediastrum* | 0.24% | - | | - |  |
| *Pediastrum duple*x | 0.24% | - | | - |  |
| *Pediastrum* duplex var. gracillimum | - | - | | 0.16% |  |
| *Scenedesmus acuminatus* | 0.91% | 0.66% | | 1.14% |  |
| *Scenedesmus bijuga* | 0.32% | 0.44% | | 1.62% |  |
| *Scenedesmus dimorphus* | 0.97% | - | | - |  |
| *Scenedesmus quadricauda* | 0.43% | - | | 0.32% |  |
| *Staurastrum* | 0.16% | - | | 0.16% |  |
| *Tetraedron* | - | 0.44% | | 2.44% |  |

Ambient; without consumer *Bellamya aeruginosa* addition.

Grazed; consumer *Bellamya aeruginosa* addition.

Table S4 Pruning and reading mapping results of sequences generated from periphyton with Ambient and Grazed group.

| Sample | Clean Reads Pairs | Clean base (bp) | Length | Q20(%) | Q30(%) | GC(%) |
| --- | --- | --- | --- | --- | --- | --- |
| Ambient -1 | 20,487,190 | 6,146,157,000 | 150;150 | 97.9;96.7 | 91.6;88.6 | 46.9;46.9 |
| Ambient -2 | 23,003,161 | 6,900,948,300 | 150;150 | 98.1;97.0 | 92.0;89.4 | 46.8;46.8 |
| Ambient -3 | 22,873,558 | 6,862,067,400 | 150;150 | 98.1;97.1 | 92.4;89.8 | 46.9;46.9 |
| Grazed-1 | 19,572,204 | 5,871,661,200 | 150;150 | 97.7;96.6 | 91.1;88.5 | 48.8;48.9 |
| Grazed -2 | 19,400,686 | 5,820,205,800 | 150;150 | 97.9;97.1 | 91.6;89.6 | 44.6;44.7 |
| Grazed -3 | 20,958,954 | 6,287,686,200 | 150;150 | 97.9;96.6 | 91.5;88.5 | 48.0;48.1 |

Ambient; without consumer *Bellamya aeruginosa* addition.

Grazed; consumer *Bellamya aeruginosa* addition.

Table S5 Functional annotation of transcriptome data in five public protein databases.

| Database | Transcripts Number (Percentage) |
| --- | --- |
| KOG | 224,297 (27.92%) |
| KEGG | 237,512 (29.56%) |
| NR | 310,915 (38.70%) |
| GO | 262,128 (32.63%) |
| Swiss-Prot | 306,207 (38.12%) |
| Unknown | 456,824 (56.86%) |
| Total | 803,356 (100%) |


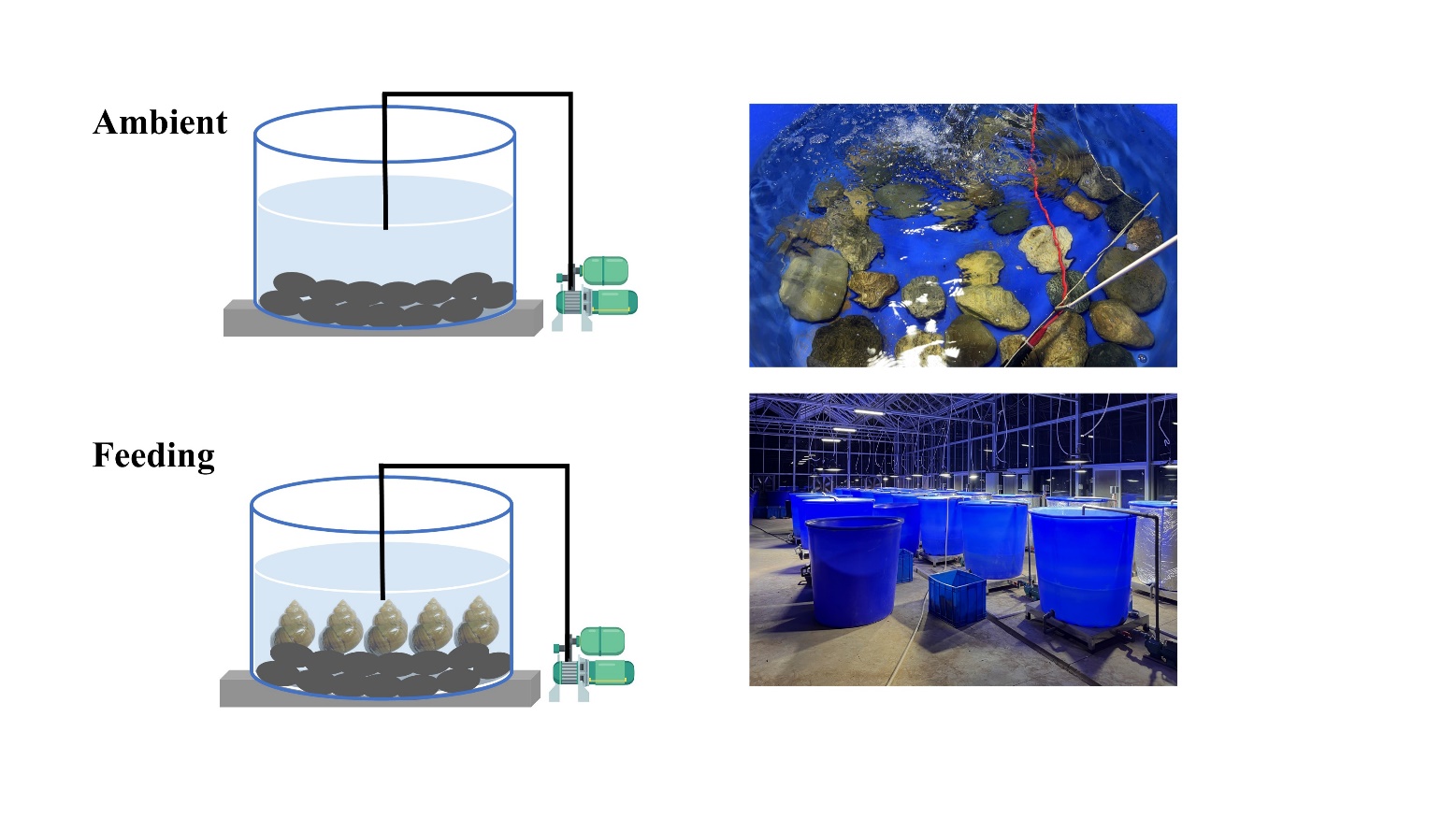


Fig. S1 The schematic diagram of experiment showed that the “Ambient”-periphyton without the consumer *Bellamya aeruginosa* addition and the “Grazed”- periphyton with the consumer *Bellamya aeruginosa*.


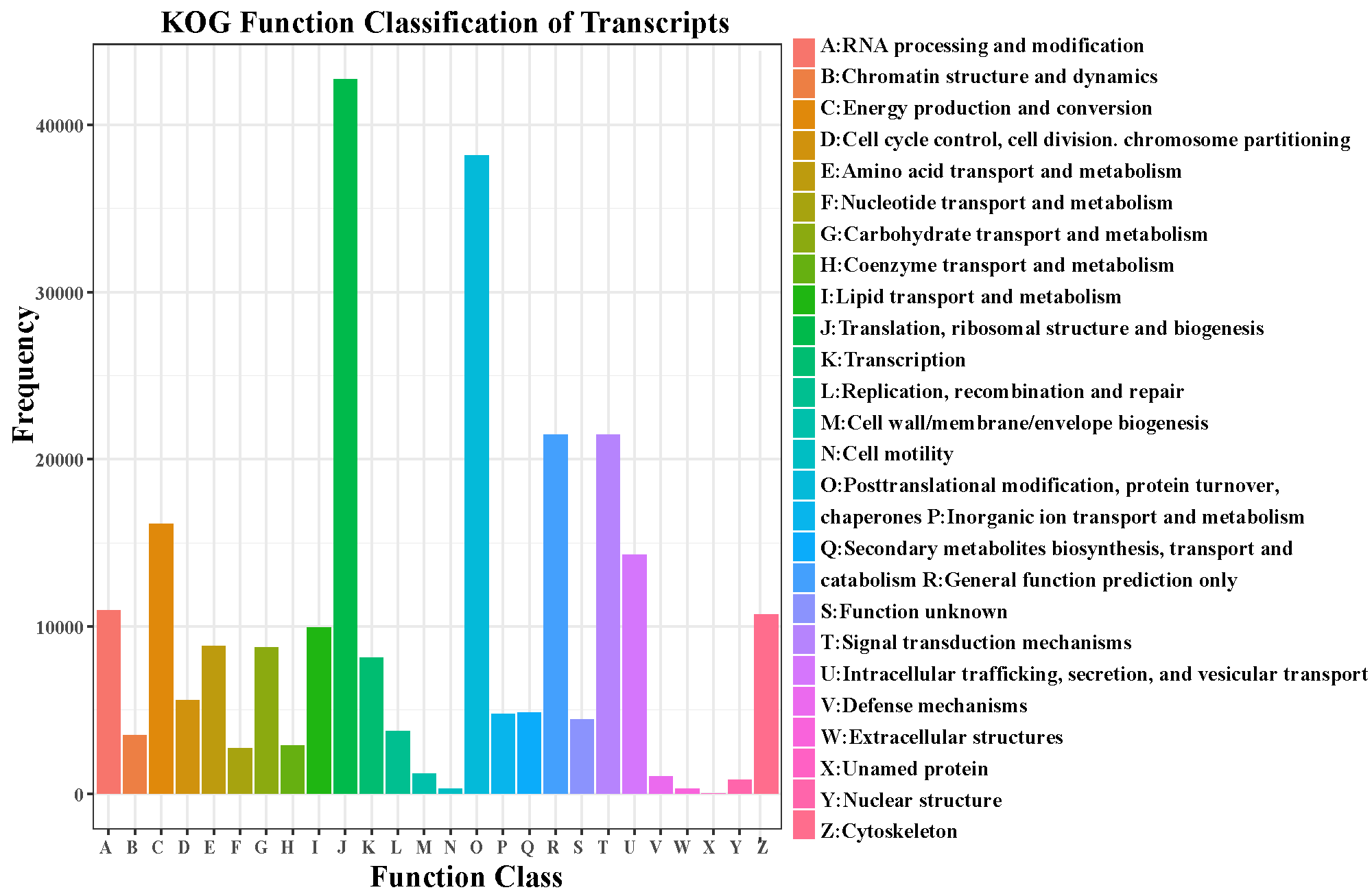


Fig. S2 KOG taxonomic diagram.


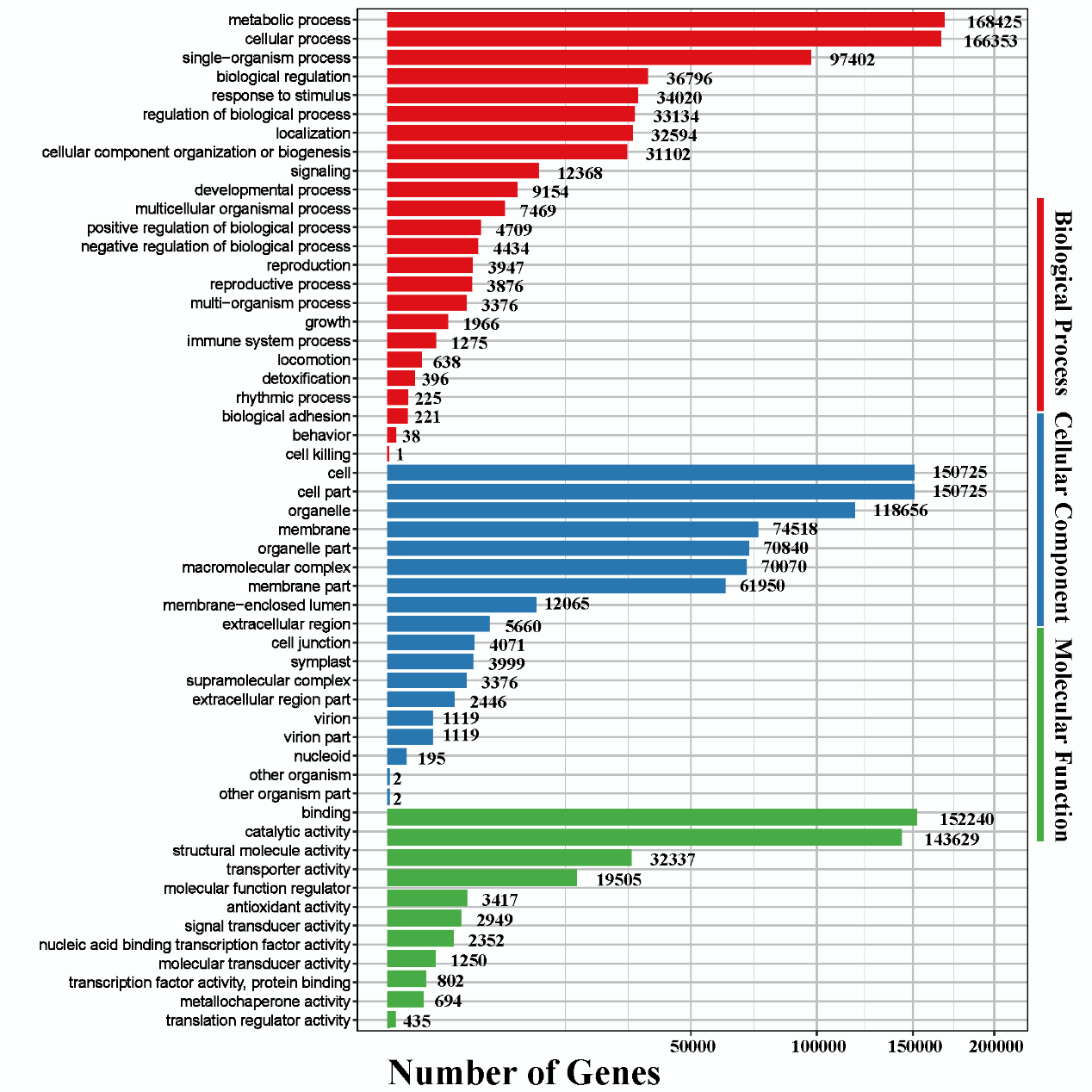


Fig. S3 GO taxonomic map. The ordinate is the GO term at the next level of the three categories of go, the x-axis is the number of transcripts annotated to the term (including the sub term of the term).


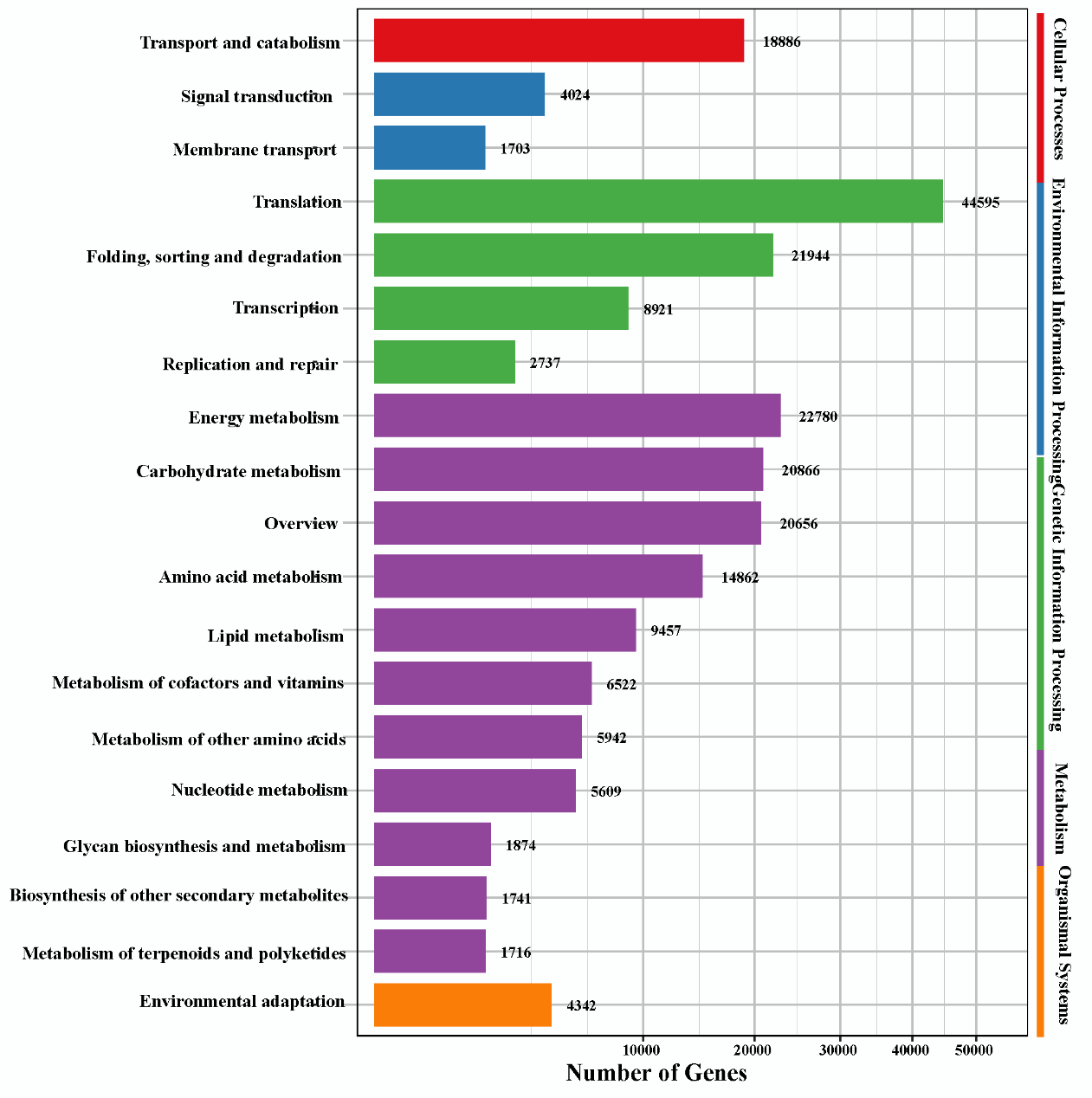
Fig. S4 KEGG taxonomic map. The ordinate is the name of KEGG pathway, the x-axis is the number of transcripts annotated to this pathway.


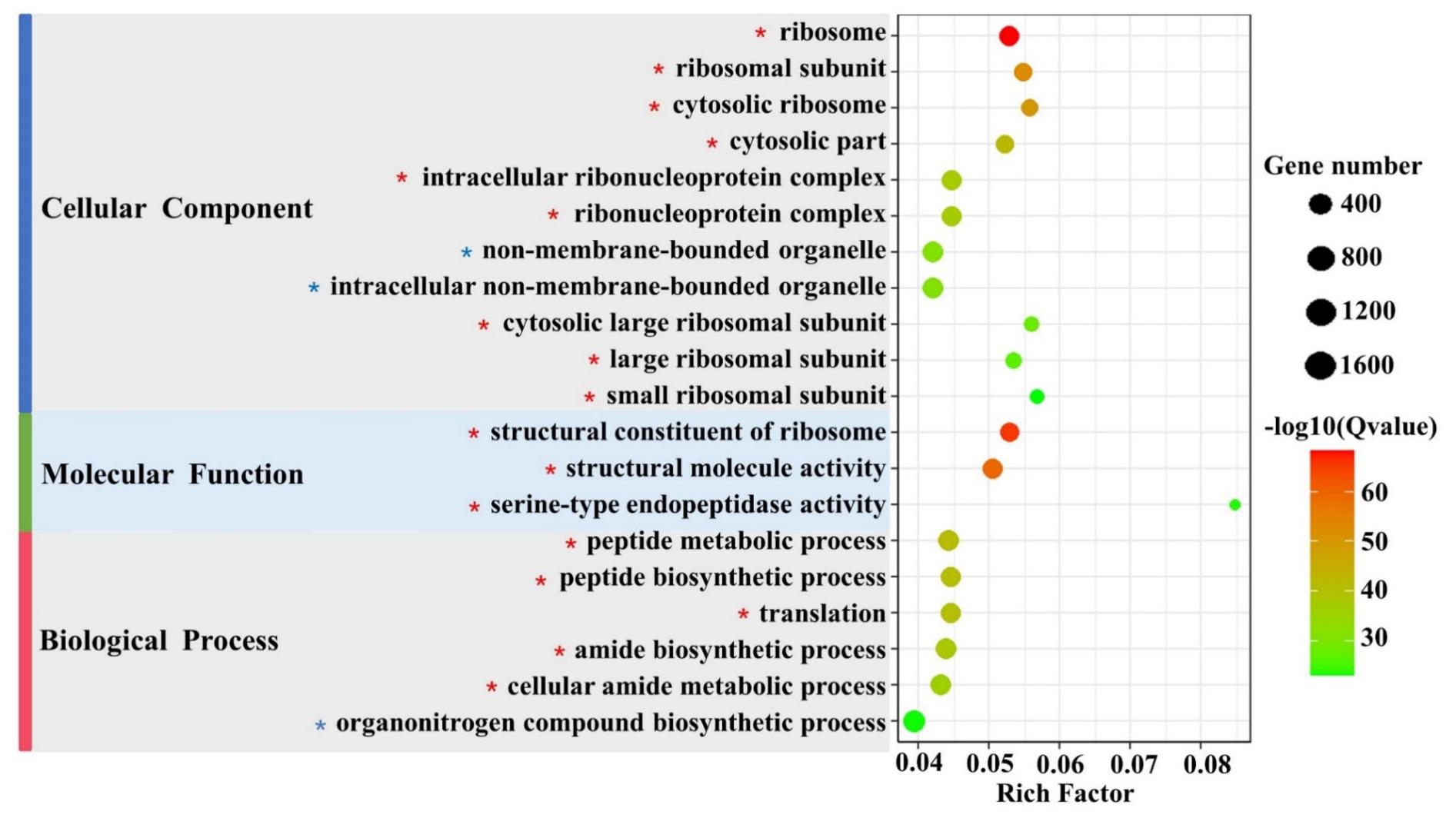


Fig. S5 Bubble diagram of differentially expressed genes (DEGs) between different treatment groups. The x-axis indicates expression changes (log) of the genes in different treatment groups while the y-axis shows the differences of gene expression. Splashes were for different genes. Black dots were genes with no significant discrepancy, red dots were genes significantly up-regulated, and blue dots were significantly down-regulated genes.


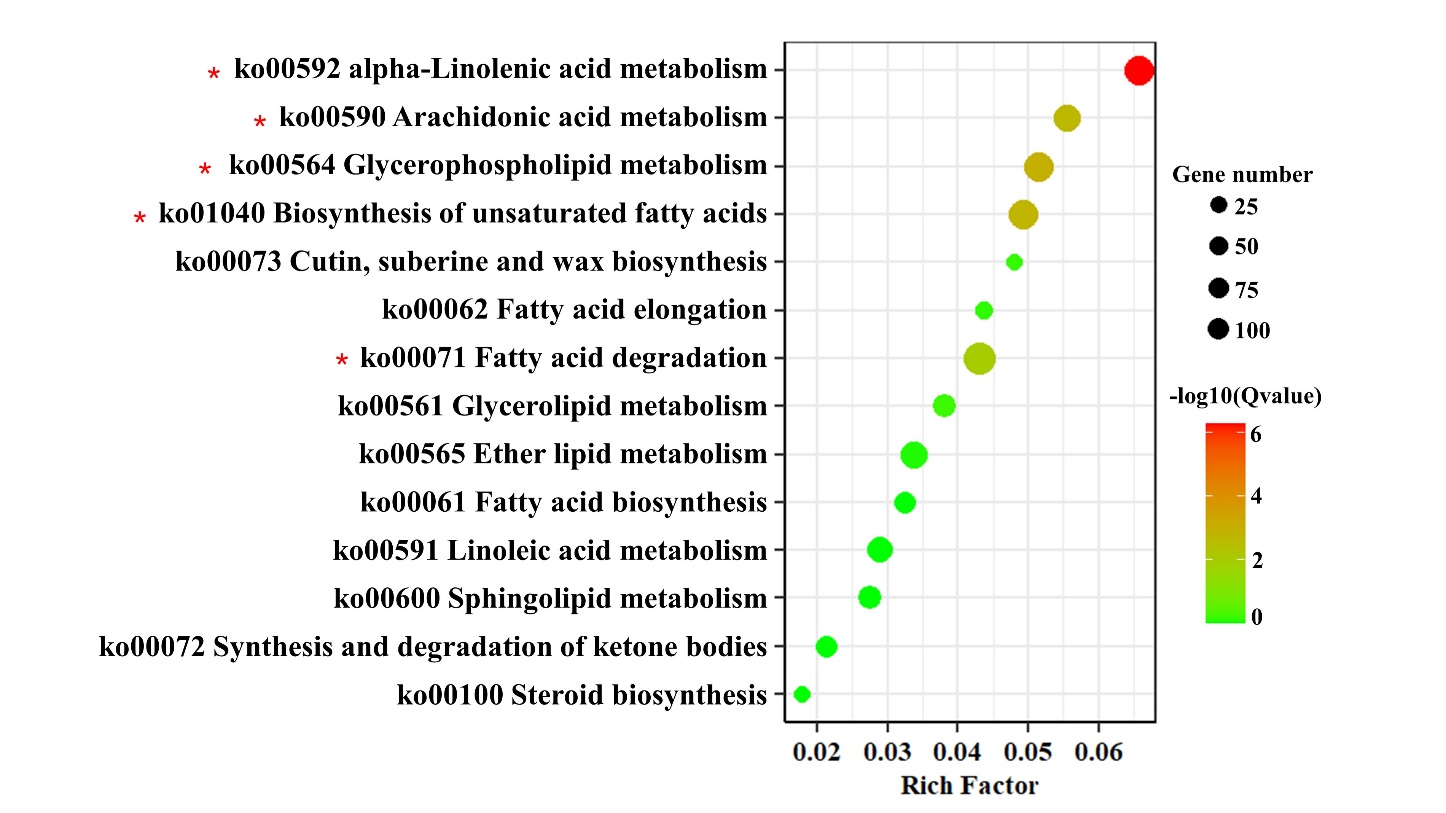


Fig. S6 Bubble diagram of Lipid metabolism enrichment analysis of differentially expressed genes (DEGs).


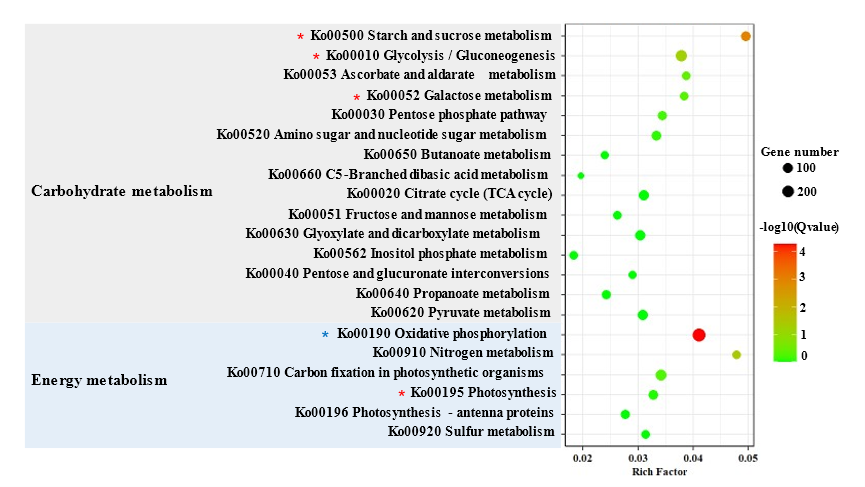


Fig. S7 Bubble diagram of Carbohydrate metabolism and Energy metabolism enrichment analysis of differentially expressed genes (DEGs). The y-axis indicates KEGG pathway and the x-axis represents the Rich Factor. The size of dots indicates the number of genes in the KEGG pathway. Red stars were pathway significantly up-regulated and blue stars were significantly down-regulated genes.
